# Editorialets

**Published:** 1910-07

**Authors:** 


### Editorialets.

"LET the doctors dissect each other; damn them,—and their ghost bill."—A. B. Davidson, candidate for Lieutenant Governor.

PROF. GEO. DOCK, late of the Tulane, has been induced to accept the Chair of Medicine in Washington University, St. Louis.

THE "RED BACK."—An enthusiastic subscriber writes: "I once dubbed the 'Red Back' the best journal in the two Republics. I now say, it is the cleanest, bravest and best published in any language."

THE ANIMAL THERAPY CO., 72 Madison Street, Chicago, will send a copy of the June Bulletin free, on request. A postal card will do it. It is full of *meat*. See Abstracts Department.

Dr. A. F. Austin, Porfessor of Chemistry, Medical Department University of Texas, has resigned, and Dr. Geo. F. Gracy, late Instructor in Chemistry, University of Pennsylvania, has been elected to succeed him.

---

Good Doctor Wanted.—A capable, experienced and sober doctor can hear of an excellent opening in an East Texas railroad town by writing the editor of this Journal (stamp enclosed). Established and good paying practice. No strings to it. Editor, Texas Medical Journal, Austin.

---

A Department of Public Health would have for its function the removal of the causes of sickness and death, and thus in a measure make the *practice of medicine* unnecessary. But—what's the use to argue with those cranks who are opposing it? "Against stupidity (and venality), even the gods are powerless." Read Irving Fisher's articles in this issue.

---

Consumptive Insane.—Two cottages for the consumptives in the (Austin) Texas Insane Asylum have been completed. They will accommodate sixty patients. Other cottages to be built to receive consumptives from the other Texas insane asylums, which have no separate accommodations, will bring the cost to $29,000.

---

Prof. J. F. Y. Paine, M. D., for nineteen years Professor of Gynecology in the (Galveston) Medical Department of the State University, retires from active work in that branch September 1st. He becomes emeritus professor, and will lecture at his convenience. The students presented him with a beautiful loving cup at the close of the session, June 1st, ult. Dr. Geo. H. Lee, of Galveston, becomes his successor September 1st. We reproduce from the *News* Dr. Singleton's address.

---

The New Officers of the A. M. A.—Dr. J. B. Murphy, of Chicago, was elected President at St. Louis. The other officers are: Dr. E. E. Montgomery, Philadelphia, First Vice-President; Dr. R. C. Caffey, Portland, Ore., Second Vice-President; Dr. W. D. Moore, St. Louis, Third Vice-President; Dr. L. E. Johnson, Washington, D. C., Fourth Vice-President; Dr. Frank Billings,

Chicago, re-elected Treasurer, and Dr. George H. Simmons, of Chicago, Secretary. Los Angeles in 1911.

Murphy defeated the great Jacoby.

---

MEDICAL DIRECTOR TABOR.—The "Red Back" is pleased to learn that its old friend Dr. Geo. R. Tabor, the popular and very efficient State Health Officer under the Lanham administration, who is now living at his old home, Bryan, Texas. has been chosen Medical Director of a strong life and accident insurance company at a big salary. Well, he'll make a good one; make a good anything where intelligence, learning, experience and good judgment count,—and where high-bred courtesy and Chesterfieldian manners are appreciated. Success to him!

---

UP AGAINST IT.—I see by the St. Louis papers that the St. Louis College of Physicians and Surgeons feel that the school has been libeled by the report of the Educational Council of the A. M. A., and that suit for $100,000 has been filed against Dr. Henry S. Pritchett, head of the Carnegie Foundation, and Dr. Abraham Flexner, its "official investigator," and Dr. Geo H. Simmons, Secretary A. M. A. and editor of its great journal. Seems to me that I recall something of a suit against the *Journal* by a chemical company in Philadelphia, a while ago.

---

OH SHAME, where is thy blush! I read with sentiments of surprise and disgust that at a banquet given by or for the Section of Surgery, A. M. A., amongst the "amusements" furnished by the caterer was a "nautch dance" by a naked woman, called "Fatima." "Clad," says the newspaper, "in her combs and garters," and that "a gray-haired doctor removed the garters." It is inconceivable that any self-respecting physician could or would be party to such shockingly immoral, disgusting performance. The "gray-haired physician" deserves the fate of Orestes—to be scourged by the Furies with whips of scorpions.

---

DEATH OF DR. EVANS, OF JEWETT.—The JOURNAL is pained to announce the death of its old friend and staunch supporter, Dr. W. T. Evans, of Jewett, Texas. He was one of the best men in the medical profession, universally esteemed and beloved by all who knew him. The town of Jewett, where he lived and labored nearly thirty years, is in mourning for the beloved physician, the exemplary citizen, the loyal friend, the Christian gentleman; the man whose heart was as big as nature and as open and generous as sunshine. He died May 28th, aged 69. He was an Alabamian. For twenty-five years he never missed a meeting of the State Association or of his local society, but he was too modest to make his presence known or felt, and was, therefore, never elected to any office. The editor of the "Red Back" will miss him, and, with thousand of friends, mourn his loss.

---

THE MEDICAL DIRECTORS' ASSOCIATION OF TEXAS was organized and held its first meeting at Dallas, May 11th. They had a rousing good time and a most satisfactory business meeting. The following officers were unanimously elected for the ensuing year: Dr. J. H. Florence, Houston, Texas, President; Dr. J. S. Lankford, San Antonio, Texas, Vice-President; Dr. M. M. Smith, Dallas, Texas, Secretary and Treasurer. The Dr. M. M. Smith mentioned is Medical Director of the Great Praetorians, whose fifteen-story building in Dallas taps the clouds, and the editor, proprietor and publisher of the one and only *Texas Medical News*, Dallas, Texas. His May number is a whizzer. It is devoted largely to a write-up of the Medical Directors and the meeting. Drop Dr. Matt a postal card and he will send you a copy; or, better, by far—for you—send him a dollar, or two, or five for the *Medical News*. It will keep you in a good humor, for Dr. Matt is nothing, if not optimistic, genial, delightful and popular.

---

DEATH OF DR. J. T. WILSON.—I reproduce the following account of the death of this distinguished Texas physician from the *Texas Medical News*:

Dr. J. T. Wilson, of Sherman, Texas, died in Washington City on May 22d, after an illness of several years' duration.

Dr. Wilson was born in Prince George county, Maryland, in the year 1845. In 1861 he joined the Patexunt Rifles and fought for the cause of the Southland. He was twice imprisoned; he served in the Signal Corps, attached to Longstreet's Corps, and he was later a member of the First Maryland Artillery, his duty being to load cannon. A number of battles were fought under General Lee, in all of which young Wilson, a boy in years, manifested the moral and physical courage that distinguished him in later life.

At the close of the war Dr. Wilson returned to his home near Harper's Ferry, where he remained until 1876, at which time he moved to Sherman, Texas, where he resided until his death. He is survived by a widow, a son and a daughter.

Dr. Wilson won for himself a distinguished position in the State of his adoption. His fame as a skilled physician, his broad culture, and his lofty traits of character gained for him the highest positions of honor and trust which the State has it in her power to bestow upon a physician. He served a number of years as Superintendent of the Asylum for the Insane at Terrell, Texas. During his incumbency there he brought to bear upon his manifold and exacting duties medical ability of an unusual order and conscientious and untiring effort, coupled with a patient, winning personality that eminently fitted him for such an arduous and delicate position. On the passage of the first law regulating the practice of medicine in Texas, Dr. Wilson was appointed by Governor Sayers as a member of the Board, which unanimously elected him President, which position he held with honor several years. Only one who was closely associated with Dr. Wilson in the discharge of duties connected with the Board of Medical Examiners can appreciate the great work which he accomplished in a field in which he and his associates labored as pioneers.

Dr. Wilson commanded the respect and admiration of his fellow practitioners of medicine in Texas. His probity, conservatism and fine executive ability inspired the medical profession to select him as President of the Texas Medical Association.

In his intercourse with the members of his profession his bearing was at all times both ethical and honorable. There was in him an entire absence of self-seeking and vulgar aggrandizement, and always the deportment of the physician who recognizes the lofty nature of his calling, and who refuses for the recompense of a few paltry dollars to subvert it to base uses.

It was as a man, however, that Dr. Wilson won from his friends, associates and the world at large the truest and most lasting encomiums. His steadfast blue eyes, beneath a lofty brow, surmounted by locks silvered by unshirked self-sacrifice, looked benignantly out on his fellowman. His gentle smile carried comfort to aching breasts. A refined and tender deference of manner characterized his intercourse with men and women alike, stamping him as a gentleman. He went forth to life's battlegrounds like another Sir Galahad to win what should be for him, the "Holy Grail"—"truth, purity and honor." That he won his goal, his life and achievements bear eloquent testimony. He fought a good fight! Honor to his memory! His armor is laid aside for the long sleep. May it be dreamless, with a glorious awakening in eternity.—*Texas Medical News*, May.

